# Advancements and Future Prospects of Energy Harvesting Technology in Power Systems

**DOI:** 10.3390/mi16080964

**Published:** 2025-08-21

**Authors:** Haojie Du, Jiajing Lu, Wenye Zhang, Guang Yang, Wenzhuo Zhang, Zejun Xu, Huifeng Wang, Kejie Dai, Lingxiao Gao

**Affiliations:** 1 College of Electric and Mechanical Engineering, Pingdingshan University, Pingdingshan 467000, China; 2629@pdsu.edu.cn (H.D.); 26109@pdsu.edu.cn (W.Z.); yg@pdsu.edu.cn (G.Y.); 231013047@e.pdsu.edu.cn (W.Z.); 231013034@e.pdsu.edu.cn (Z.X.); 231013059@e.pdsu.edu.cn (H.W.); 2 School of Mechanical Engineering, Hebei University of Technology, Tianjin 300401, China; lujiajing0101@163.com; 3 Key Laboratory of Advanced Intelligent Protective Equipment Technology, Ministry of Education, Hebei University of Technology, Tianjin 300401, China

**Keywords:** energy harvesting, power systems, the current research status, the future development trajectory

## Abstract

The electric power equipment industry is rapidly advancing toward “informationization,” with the swift progression of intelligent sensing technology serving as a key driving force behind this transformation, thereby triggering significant changes in global electric power equipment. In this process, intelligent sensing has created an urgent demand for high-performance integrated power systems that feature compact size, lightweight design, long operational life, high reliability, high energy density, and low cost. However, the performance metrics of traditional power supplies have increasingly failed to meet the requirements of modern intelligent sensing, thereby significantly hindering the advancement of intelligent power equipment. Energy harvesting technology, characterized by its long operational lifespan, compact size, environmental sustainability, and self-sufficient operation, is capable of capturing renewable energy from ambient power sources and converting it into electrical energy to supply power to sensors. Due to these advantages, it has garnered significant attention in the field of power sensing. This paper presents a comprehensive review of the current state of development of energy harvesting technologies within the power environment. It outlines recent advancements in magnetic field energy harvesting, electric field energy harvesting, vibration energy harvesting, wind energy harvesting, and solar energy harvesting. Furthermore, it explores the integration of multiple physical mechanisms and hybrid energy sources aimed at enhancing self-powered applications in this domain. A comparative analysis of the advantages and limitations associated with each technology is also provided. Additionally, the paper discusses potential future directions for the development of energy harvesting technologies in the power environment.

## 1. Introduction

With the emergence of the Internet of Things (IoT) and artificial intelligence, the concept of sensor networks—a cornerstone technology—has become deeply ingrained in public consciousness. Research indicates that by 2025, over 30 billion devices will be connected via IoT [1]. Grid digitization necessitates the deployment of a substantial number of sensors to enable intelligent monitoring of grid conditions [2,3]. Although the power consumption of sensors can be reduced to the milliwatt level, the issue of energy supply for a large number of sensor nodes remains a critical challenge [4]. Currently, the operation and maintenance of sensors used in power grid equipment condition monitoring require substantial human and material resources. Conventional batteries, which serve as the primary power source for such monitoring systems, present several limitations, including a short service life, difficulties associated with replacement, and potential environmental pollution [5,6,7,8]. In recent years, the concept of environmentally friendly, self-powered, low-power devices that harness energy from the surrounding environment has increasingly attracted the attention of scientists. Consequently, micro-energy harvesting technology has emerged as a timely solution to meet growing energy demands [9,10].

**Figure 1:**
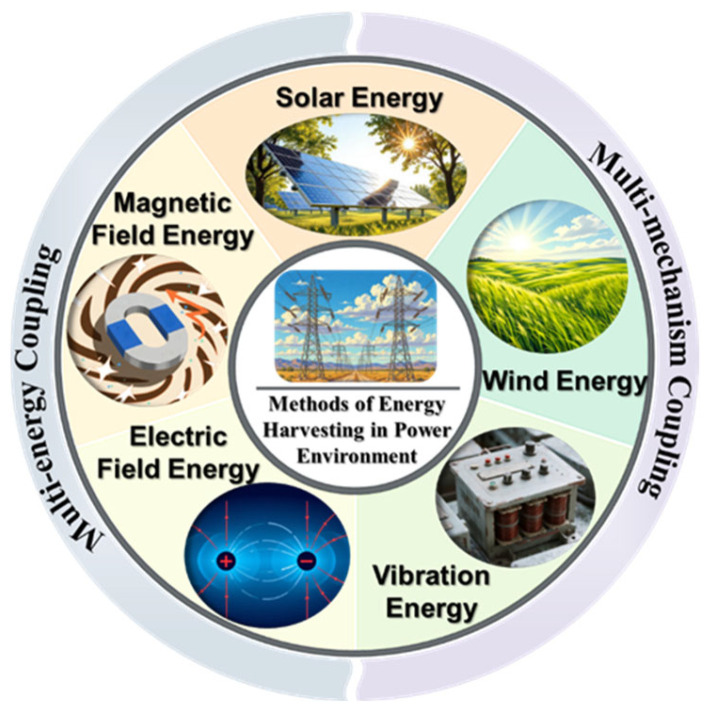
The forms of energy present in the power environment and the corresponding collection methods.

Realizing in situ energy supply for sensors within the power environment through the collection of renewable energy present in that environment represents a disruptive technology in the new energy revolution [11,12,13,14,15]. At present, the energy harvesting technologies in the power environment mainly include magnetic field energy harvesting [16,17], electric field energy harvesting [18,19], vibration energy harvesting [20,21], wind energy harvesting [22,23], and solar energy harvesting [24,25], as shown in Figure 1. To systematically characterize these energy harvesting technologies, Table 1 presents a unified taxonomy based on two key dimensions: energy sources (e.g., magnetic, mechanical, and radiant) and conversion mechanisms (e.g., electromagnetic induction and the piezoelectric effect). This classification framework serves as a foundational structure for the subsequent analysis and discussion. This paper presents a comprehensive review of the current development status of energy harvesting technologies within the power environment. It outlines recent advancements in magnetic field energy harvesting, electric field energy harvesting, vibration energy harvesting, wind energy harvesting, and solar energy harvesting. Furthermore, it explores multi-physical mechanism coupling and multi-energy integration approaches in self-powered power systems and provides a comparative analysis of the advantages and limitations associated with each technology. Additionally, the paper discusses potential future directions for the development of energy harvesting technologies in power-related applications.

## 2. Magnetic Field Energy Harvesting Technology

In high-voltage power cable environments, the surrounding magnetic field is significantly stronger compared to that in the air. Jiameng Yang et al. measured the magnetic induction intensity of the pole bus reactor at the ±660 kV converter station in Jiaodong under full load conditions. The maximum magnetic induction intensity recorded at a distance of 1.5 m above ground level, outside the pole grid, reached 353 μT [26]. Therefore, harnessing magnetic field energy from power environments to supply electricity to sensors represents a highly promising solution [27]. Magnetic field energy harvesting is a technology that captures energy from magnetic fields and converts it into a usable form [28,29]. At present, the utilization of magnetic field energy in power systems can generally be classified into two main categories: direct conversion technology and indirect conversion technology.

### 2.1. Direct Magnetic Field Energy Harvesting Technology

**Figure 2:**
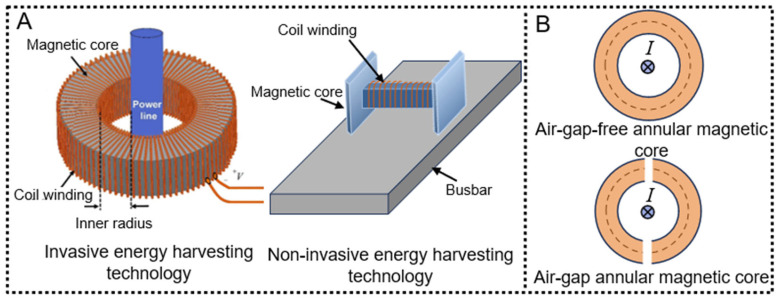
Classification of direct magnetic field energy conversion technologies: (**A**) Two categories of direct magnetic field energy conversion technologies. Reprinted with permission from [30]; (**B**) Two types of invasive energy harvesting methods.

The core principle of direct conversion technology is grounded in Faraday’s law of electromagnetic induction. When a coil is exposed to a varying magnetic flux or when magnetoelectric materials are employed, an induced voltage is generated in response to changes in the magnetic field. This phenomenon enables the direct conversion of magnetic energy into electrical energy. Direct conversion technology can be categorized into two primary types according to the installation method of the energy harvesting module: invasive and non-invasive (Figure 2A). The fundamental distinction between these two lies in whether the original conductor needs to be physically disconnected, wound around, or internally embedded. Invasive energy harvesting operates in a manner similar to conventional current transformers, where energy is obtained through insertion-based coupling with transmission lines or AC buses. The invasive type can be further classified into two subcategories: cable-clamping configurations with and without an air gap ring core (Figure 2B).

**Figure 3:**
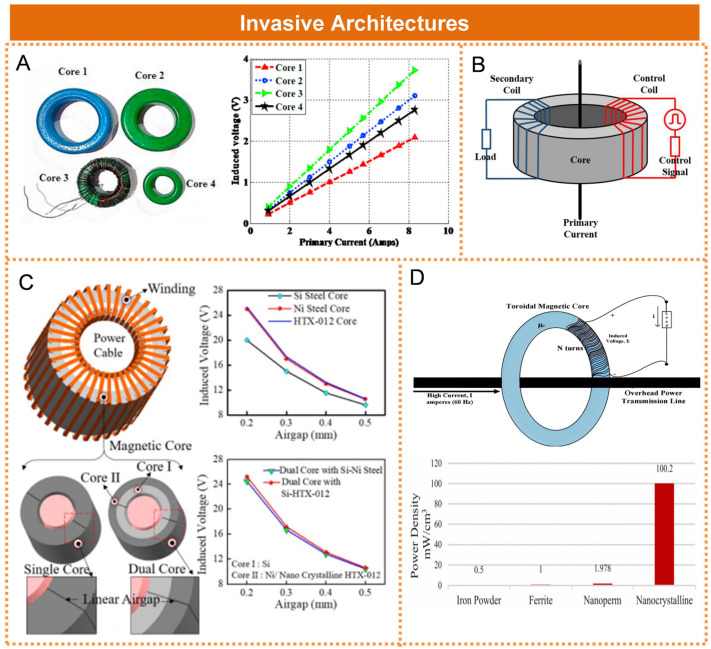
Invasive magnetic field energy harvesting architectures: (**A**) Architecture based on cable-clamping air-gapless toroidal magnetic core. Reprinted with permission from [31]. (**B**) Scheme introducing an artificial magnetic field. Reprinted with permission from [32]. (**C**) Architecture based on a cable-clamping toroidal double magnetic core with an air gap. Reprinted with permission from [33]. (**D**) Energy harvesting system utilizing toroidal magnetic core induction from overhead power lines. Reprinted with permission from [34].

A. A. Gaikwad et al. developed an inductive energy harvester utilizing a cable-clamping, air-gapless toroidal core configuration, as illustrated in Figure 3A. The researchers conducted a systematic investigation into the impact of iron cores with identical permeability but varying dimensions on the output voltage, highlighting the critical role of core size optimization in achieving efficient energy conversion [31]. To address the limitation in power extraction associated with conventional magnetic energy harvesters—specifically, the constraint imposed by the core’s maximum magnetic flux density—Yuan Zhuang et al. proposed a novel approach involving the introduction of an artificial magnetic field through the addition of a control coil, as illustrated in Figure 3B. A power management circuit was employed to store the energy harvested by the control coil and subsequently fed it back into the system to generate a counter magnetic field. The experimental results demonstrated that the proposed energy harvester achieved an average power output of 283 mW when applied to a 50 Hz, 10 Arms power line. This represented a 45% improvement compared to the performance of a device without a control coil [32]. Sarbajit Paul et al. proposed a cable-clamping, air-gap ring-shaped dual-iron core magnetic field energy harvesting device, as illustrated in Figure 3C. Research indicated that the core loss of the dual-iron core model was significantly lower compared to that of the silicon steel single-iron core model [33]. Syed Ahmed Ali Najafi et al. developed a high-efficiency magnetic field energy harvester utilizing a nanocrystalline toroidal cut-core configuration, as illustrated in Figure 3D [34]. The researchers conducted a comprehensive analysis of core material properties, emphasizing the pivotal role of nanocrystalline alloys in achieving a record power density of 100.2 mW/cm^3^ and stable thermal performance. Jiajia Zhang et al. developed a magnetic field energy harvesting device utilizing a double-loop toroidal magnetic core. By modulating the opening angles of both the inner and outer loops, a segmented magnetic circuit was constructed, which significantly reduced the magnetic reluctance and minimized flux leakage at the air gap [35].

**Figure 4:**
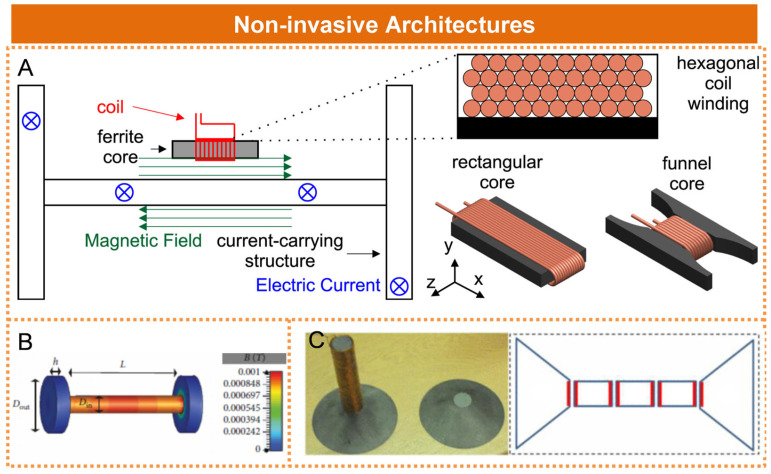
Non-invasive magnetic field energy harvesting architecture: (**A**) Collecting magnetic field energy from current-carrying structures. Reprinted with permission from [36]. (**B**) Independent I-type magnetic core. Reprinted with permission from [37]. (**C**) Bow-tie magnetic core structure. Reprinted with permission from [38].

Non-intrusive energy harvesting refers to the placement of the energy harvesting module outside the target device, enabling energy acquisition through external coupling without interfering with the device’s internal structure or operation. Steven W. Wright et al. proposed an inductive approach for harvesting magnetic field energy from current-carrying structures, as illustrated in Figure 4A [36]. The experimental results indicated that, in the presence of a spatially distributed 800 Hz, 20 A current, a core and coil with dimensions of 40 mm × 20 mm × 2 mm were capable of delivering more than 1 mW of rectified power to a capacitor. Hao Wang et al. designed an independent I-shaped core to capture the electromagnetic energy produced by large alternating currents, as illustrated in Figure 4B. The addition of a pair of flux collectors at both ends of the rod-shaped core enables more effective guidance of magnetic flux [37]. Sheng Yuan et al. employed a bow-tie-shaped magnetic core design to improve energy conversion efficiency, as illustrated in Figure 4C [38]. The experimental results demonstrated that under a magnetic flux density of 7 μTrms, the power density achieved was 1.86 μW/cm^3^. Although the coupling coefficient of non-intrusive energy harvesting is lower than that of intrusive energy harvesting, and the harvested power is relatively limited, the installation of non-intrusive energy harvesting devices does not compromise the integrity of the original system structure. Furthermore, this approach offers advantages in terms of operational convenience and maintenance cost efficiency.

Table 2 presents a summary of recent research on direct magnetic field energy harvesting technology. The invasive magnetic field energy harvesting system is capable of direct contact with the target object, thereby achieving a higher power density. This makes it particularly suitable for applications requiring high energy output. Additionally, the system demonstrates high energy conversion efficiency due to direct contact, which helps minimize energy loss during the harvesting process. However, the following disadvantages should be noted. Complex installation: The installation process is technically demanding and associated with high equipment requirements, posing potential risks. Challenging maintenance: Once implanted, maintenance and component replacement are logistically difficult and technically complicated. The advantages of non-invasive magnetic field energy harvesting include convenient installation, as the process is simple and requires minimal setup. Additionally, maintenance is straightforward, with equipment that is easy to service and replace. The disadvantages include low power density due to the lack of direct contact with the target object, which results in lower energy conversion efficiency, making it more suitable for applications with low power requirements. Another limitation is its susceptibility to signal interference from external environmental factors, which can affect the stability and reliability of the energy harvesting process.

### 2.2. Indirect Magnetic Field Energy Harvesting Technology

**Figure 5:**
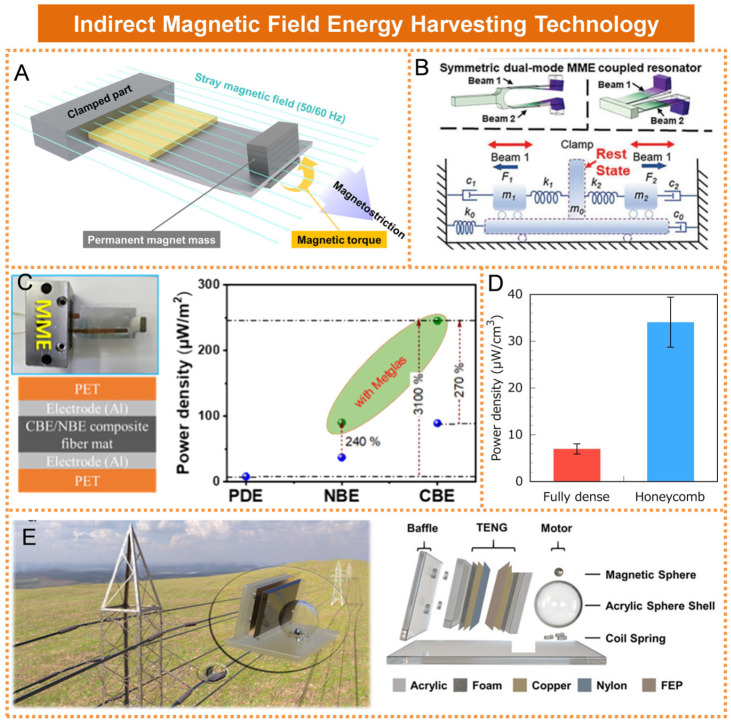
Indirect magnetic field energy harvesting technologies: (**A**) Magneto-electromechanical generator based on PZT-5H and Ni. Reprinted with permission from [46]. (**B**) Symmetric mechanical coupling dual-mode MME energy harvester. Reprinted with permission from [47]. (**C**) MME based on piezoelectric ferromagnetic (PVDF/BZT-BCT-ferrite) electrospun fiber composites. Reprinted with permission from [48]. (**D**) Output performance of honeycomb-structured, magnetostrictive Fe_52_-Co_48_ alloy. Reprinted with permission from [49]. (**E**) Triboelectric nanogenerator based on a rotating magnetic sphere. Reprinted with permission from [50].

Indirect magnetic field energy harvesting technology is an energy conversion method that employs specialized materials or structures to convert alternating magnetic field energy into mechanical energy [45]. This mechanical energy is subsequently transformed into electrical energy through integrated mechanisms such as piezoelectric and triboelectric conversion. Currently, the most widely adopted technology is vibration energy harvesting based on cantilever beams. Xin He et al. proposed a magnetoelectric generator composed of PZT-5H and Ni, as illustrated in Figure 5A [46]. The output performance of the magnetoelectric (MME) generator was improved by optimizing the ratio of the length of the piezoelectric material to that of the magnetostrictive material, as well as the total thickness of the magnetostrictive layer. Zhonghui Yu et al. proposed a symmetrical mechanical-coupled dual-mode magnetoelectric (MME) energy harvester, as illustrated in Figure 5B [47]. When subjected to a weak alternating magnetic field (Hac = 4 Oe) at a frequency of 60 Hz, the MME energy harvester operating in symmetrical dual mode achieved a peak output power of 72 mW (RMS: 9 mW), representing an increase of 437% compared to traditional single-mode MME generators. Durga Prasad Pabba et al. developed a magnetoelectric generator (MME) based on piezoelectric ferromagnetic (PVDF/BZT-BCT-ferrite) electrospun fiber composites, as illustrated in Figure 5C [48]. Under an alternating magnetic field of 50 Hz and 6 Oe, the device achieved an output voltage of 6.2 V and a power density of 88.7 μW/m^2^, representing an enhancement of 385% compared to traditional MMEs. Hiroki Kurita et al. developed a cellular-structured magnetostrictive Fe_52_-Co_48_ alloy using additive manufacturing techniques, which exhibited a power density 4.7 times higher than that of the fully dense counterpart, as illustrated in Figure 5D [49].

Most of the earlier indirect magnetic energy harvesting technologies involved coupling magnetic materials with piezoelectric materials to enable magnetic–mechanical–electrical energy conversion. In recent years, alongside the advancement of triboelectric nanogenerator (TENG) technology, researchers have started to investigate the integration of magnetic materials with triboelectric mechanisms for the purpose of achieving indirect magnetic energy harvesting. Xu Jin et al. developed a triboelectric nanogenerator based on a rotational magnetic ball to capture magnetic energy from transmission lines, as illustrated in Figure 5E [50]. Under the influence of the alternating magnetic field produced by the transmission line, the magnetic ball rotated within the spherical shell. The centrifugal force generated during this rotation drove the triboelectric unit, enabling electricity generation. A single triboelectric unit achieved an open-circuit voltage of up to 1.5 kV and an output power of 6.67 mW. Zhihao Yuan et al. developed a swing-structured triboelectric nanogenerator designed for magnetic energy harvesting. The device achieved a maximum peak power output of 0.78 mW under a load resistance of 30 MΩ [51].

A comparative summary of the research on indirect magnetic field energy harvesting technology is presented in Table 3. Magnetic–mechanical–electrical energy harvesters based on the magneto-toroidal effect and piezoelectric effect are capable of simultaneously capturing magnetic field energy and ambient vibration energy, which has made them a focal point in current micro-energy device research. However, conventional structures such as cantilever beams, once installed, are limited to collecting magnetic field energy from a single direction. This constraint hinders the efficient utilization of stray magnetic field energy that exists in various directions within real-world electromagnetic environments. Therefore, omnidirectional and multimodal magnetic–mechanical–electrical energy harvesters represent promising research directions for future development [52].

## 3. Electric Field Energy Harvesting Technology

**Figure 6:**
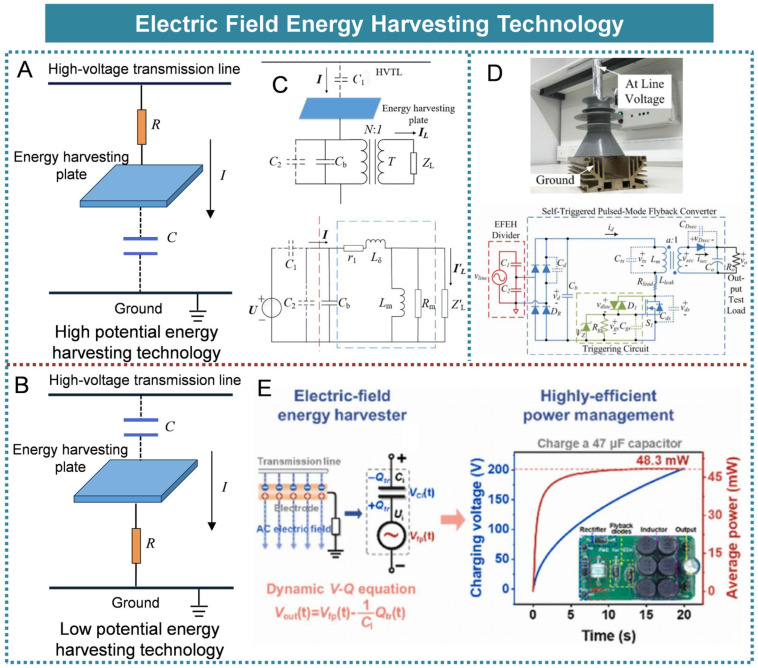
Electric field energy harvesting technologies: (**A**) High-potential energy harvesting method. (**B**) Low-potential energy harvesting method. (**C**) A method for collecting spatial electric field energy by utilizing the impedance conversion characteristics of transformers and the reactive power compensation characteristics of capacitors. Reprinted with permission from [59]. (**D**) A self-triggered pulsed-mode flyback converter for electric field energy harvesting. Reprinted with permission from [60]. (**E**) A scheme for maximizing energy transmission efficiency by managing charge transfer at peak voltage. Reprinted with permission from [61].

Electric field energy is a prevalent form of environmental energy within power systems, making the extraction of electricity from electric fields a focal point for numerous research institutions. In recent years, significant progress has been achieved in this field. The energy source harnessed through electric field energy harvesting technology is the electric field itself, and this method is also referred to as electric field induction-based energy harvesting. Depending on the installation location of the energy harvesting electrodes and the specific application context, electric field induction energy harvesting can be categorized into two types: high-potential and low-potential configurations. The energy acquisition principles of high-potential and low-potential electric field induction are fundamentally the same, as both methods derive energy from the displacement current generated by the parasitic capacitance between the transmission line and the ground. The electrodes designed for high-potential energy extraction are commonly positioned in proximity to high-voltage circuits or regions characterized by elevated electrical potential (as illustrated in Figure 6A). In contrast, electrodes intended for low-potential energy extraction are generally situated near the ground or within areas exhibiting potentials close to that of the earth (as depicted in Figure 6B).

Zong Li et al. proposed a method for harvesting spatial electric field energy based on the impedance transformation property of the transformer and the reactive power compensation capability of the capacitor, as illustrated in Figure 6C [59]. Experimental results demonstrated that under conditions where the supply voltage was 50 kV, the stray capacitance was 14 pF, the compensation capacitance was 0.86 nF, and the load resistance was 1 kΩ; the energy harvesting system achieved a power output of 340 mW. To address the challenge of weak capacitive coupling between power lines and electric field energy harvesting systems, Juan Carlos Rodriguez et al. proposed the implementation of a self-triggered, discontinuous conduction mode flyback converter for high-to-low voltage conversion, as illustrated in Figure 6D [60]. Experimental results indicated that this method was capable of consistently extracting 20 mW of continuous power from a 12.7 kV power line. To address the challenge of limited output power in conventional low-potential electric field energy harvesting technologies, Dongyang Hu et al. proposed a strategy aimed at maximizing energy transfer efficiency through controlled charge transfer at peak voltage, as illustrated in Figure 6E [61]. Experimental results indicated that the average output power of the low-potential electric field energy harvester was significantly enhanced, increasing by 1122 times (from 0.043 mW to 48.3 mW).

A summary of the technology related to electric field energy harvesting is presented in Table 4. The power harvested through the capacitive voltage division method is significantly limited. To satisfy practical application requirements, a large spatial capacitor must be employed, which poses challenges in installation and may compromise the insulation clearance of power equipment.

## 4. Wind Energy Harvesting Technology

As a manifestation of atmospheric circulation, wind energy represents one of the most significant components within sustainable energy systems, characterized by its abundant renewable potential [74]. High-voltage transmission towers are typically constructed on mountainsides or mountain peaks, where wind energy resources are abundant. However, conventional electromagnetic wind turbines, which are based on asynchronous or synchronous generator technologies, tend to be large in size, expensive, and challenging to install. While they are well-suited for large-scale wind power integration into the electrical grid, they are not ideal for powering distributed sensors within the Internet of Things (IoT) networks. In recent years, small-scale wind energy harvesting devices—characterized by high efficiency, flexible deployment, adaptable structural design, and low cost—have garnered significant attention within the field of wind energy collection [75,76,77,78,79,80].

**Figure 7:**
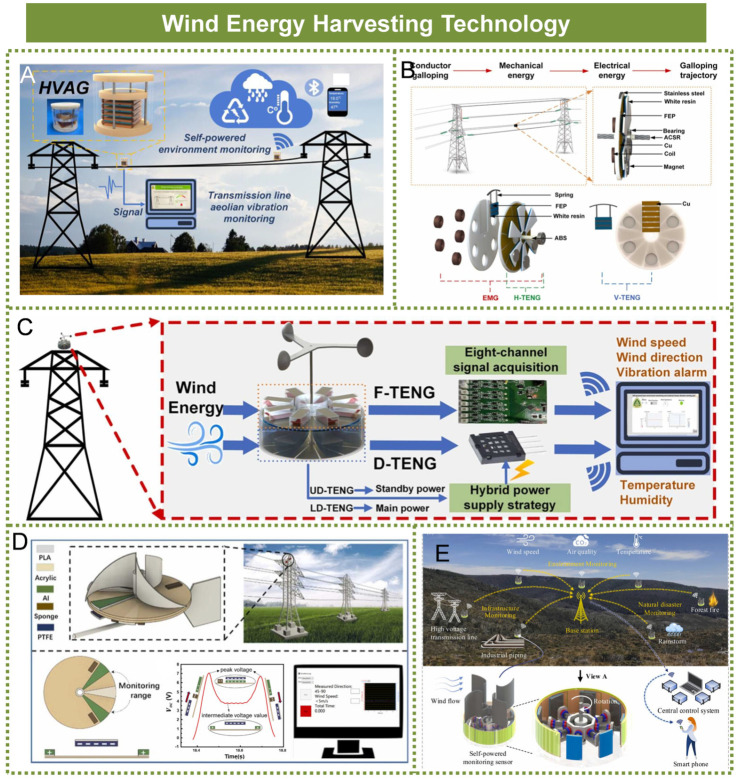
Wind energy harvesting technologies: (**A**) Triboelectric–electromagnetic hybrid wind energy harvesting system. Reprinted with permission from [81]. (**B**) Self-powered transmission line galloping monitoring system. Reprinted with permission from [82]. (**C**) Multi-directional and broadband wind energy harvesting device. Reprinted with permission from [83]. (**D**) Self-powered overhead line wind speed and direction acquisition system. Reprinted with permission from [84]. (**E**) Wind energy harvesting device with a self-regulating strategy. Reprinted with permission from [85].

Based on the triboelectric–electromagnetic hybrid working principle, Sihang Gao et al. integrated a multi-layer elastic structure triboelectric nanogenerator (ME-TENG) with a dual electromagnetic generator (EMG) to efficiently harvest wind-induced vibration energy from transmission lines [81]. Furthermore, they developed a self-powered monitoring system for transmission line vibration status, as illustrated in Figure 7A. Sihang Gao et al. designed a hybrid energy harvesting device that integrated triboelectric and electromagnetic mechanisms, specifically tailored to address the low-frequency and large-amplitude self-excited vibrations of transmission lines induced by wind. This innovative device enabled efficient self-powered real-time monitoring of galloping conditions in transmission lines, as illustrated in Figure 7B [82]. Sihang Gao et al. developed a self-powered multi-directional wind-driven triboelectric nanogenerator (SMW-TENG) capable of harvesting broadband wind energy and applicable for monitoring weather conditions, including wind speed and direction, as well as assessing the vibration status of transmission lines within power transmission systems [83]. The device demonstrated efficient energy harvesting performance across a wind speed range of 3.7 to 16.3 m per second, as illustrated in Figure 7C. Xiaolong Tang et al. developed a self-powered wind speed and direction sensor based on a triboelectric nanogenerator, designed specifically for monitoring the wind conditions and vibration states of overhead transmission lines, as illustrated in Figure 7D. Experimental results indicated that the device was capable of accurately measuring wind speeds within the range of 1.7 m/s to 6.7 m/s and effectively detecting the vibration status of the transmission lines [84]. HongXiang Zou et al. developed a wind energy harvesting device incorporating a self-regulating strategy to efficiently capture ambient wind energy across varying wind speeds [85]. Utilizing this device, they further constructed a self-powered wireless wind speed measurement system, which is applicable to autonomous infrastructure monitoring, as illustrated in Figure 7E.

Small-scale wind energy harvesting devices have exhibited significant advantages in the development of localized energy supply systems. However, their performance across varying wind directions and a wide range of wind speeds remains limited, and structural stability for long-term outdoor deployment requires further improvement. Table 5 presents a detailed summary of the advantages and disadvantages associated with the overall performance of small-scale wind energy systems.

## 5. Solar Energy Harvesting Technology

Solar energy harvesting technology primarily converts light energy into electrical energy through the use of photovoltaic panels, which can then be utilized to power sensors. Given the temporal fluctuations in light intensity, solar panels are commonly integrated with batteries in practical applications to constitute a complete solar power supply system.

**Figure 8:**
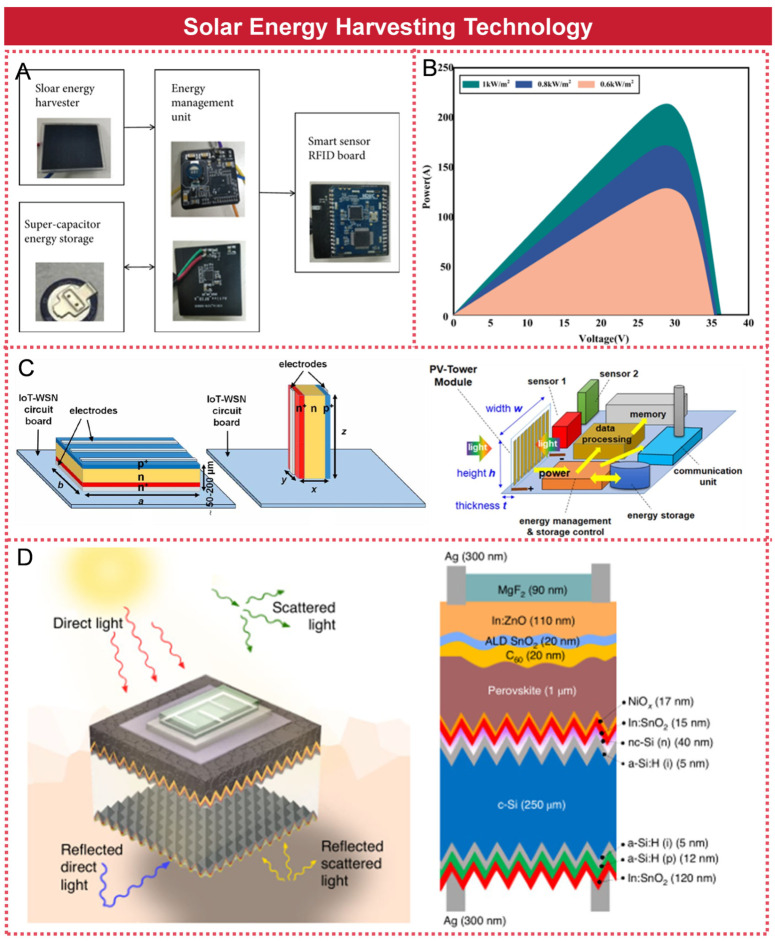
Solar energy harvesting technologies: (**A**) Self-powered intelligent sensing system for transmission lines based on solar energy. Reprinted with permission from [86]. (**B**) Transmission line equipment health status monitoring system based on self-powered sensors. Reprinted with permission from [87]. (**C**) Solar cell with a side wall light energy absorption function. Reprinted with permission from [88]. (**D**) Solar cell with light energy absorption function on both sides. Reprinted with permission from [89].

Wanli Sun et al. developed a solar-powered, self-sustaining intelligent sensing system for transmission lines, as illustrated in Figure 8A. This system comprised a solar panel-based energy harvester, a micro-energy management unit, an intelligent sensor module, and a supercapacitor [86]. It was capable of emitting a 15 mA current pulse every 30 min, with each pulse lasting 730 ms. Beibei Guo et al. proposed a deep perception system for monitoring the health status of transmission line equipment by utilizing self-powered sensors, as illustrated in Figure 8B. The integration of self-powered sensors into the deep sensing system enabled optimization of both the transmission line network model and the data transmission compression algorithm. Experimental results demonstrated that photovoltaic cells can provide stable and sufficient power to support sensor operations, thereby extending the service life of the sensor nodes [87]. Ari Bimo Prakoso et al. proposed a photovoltaic solar cell capable of absorbing light energy not only from the top surface but also from the large side walls, thereby achieving a compact footprint and high output power, as illustrated in Figure 8C. The output power density of a series-connected array of 10 photovoltaic cells, covering a total area of 0.85 square centimeters, reaches 78.5 milliwatts per square centimeter—approximately 80 times higher than that of conventional solar cells [88]. Michele De Bastiani et al. proposed a bifacial perovskite/silicon tandem solar cell, which was capable of absorbing sunlight from the front as well as reflected light from the environment at the rear, as illustrated in Figure 8D. The device achieved a power conversion efficiency exceeding 25%, with a power density reaching up to 26 milliwatts per square centimeter [89].

Solar power generation technology offers significant economic and environmental benefits and is based on a well-established technological foundation. It is primarily suited for outdoor areas with ample sunlight. However, solar panels tend to be large and heavy, with relatively low photovoltaic conversion efficiency. Additionally, they are unable to function effectively in adverse weather conditions, such as cloudy or rainy days, and do not operate at night. Regular maintenance is also required to ensure optimal performance.

## 6. Vibration Energy Harvesting Technology

Vibration energy is a form of energy that is commonly present in various power environments. Harnessing vibration energy for electricity generation can effectively reduce dependence on traditional batteries, decrease overall energy consumption, and mitigate environmental pollution. As a result, vibration energy harvesting has emerged as a key research focus within the field of energy harvesting [90,91,92,93]. Based on the type of energy involved and the underlying energy conversion mechanisms, mechanical vibration energy harvesting technologies can generally be categorized into three primary types: piezoelectric energy harvesting [94,95,96,97], electromagnetic energy harvesting [98,99,100], and triboelectric energy harvesting [101,102,103,104].

**Figure 9:**
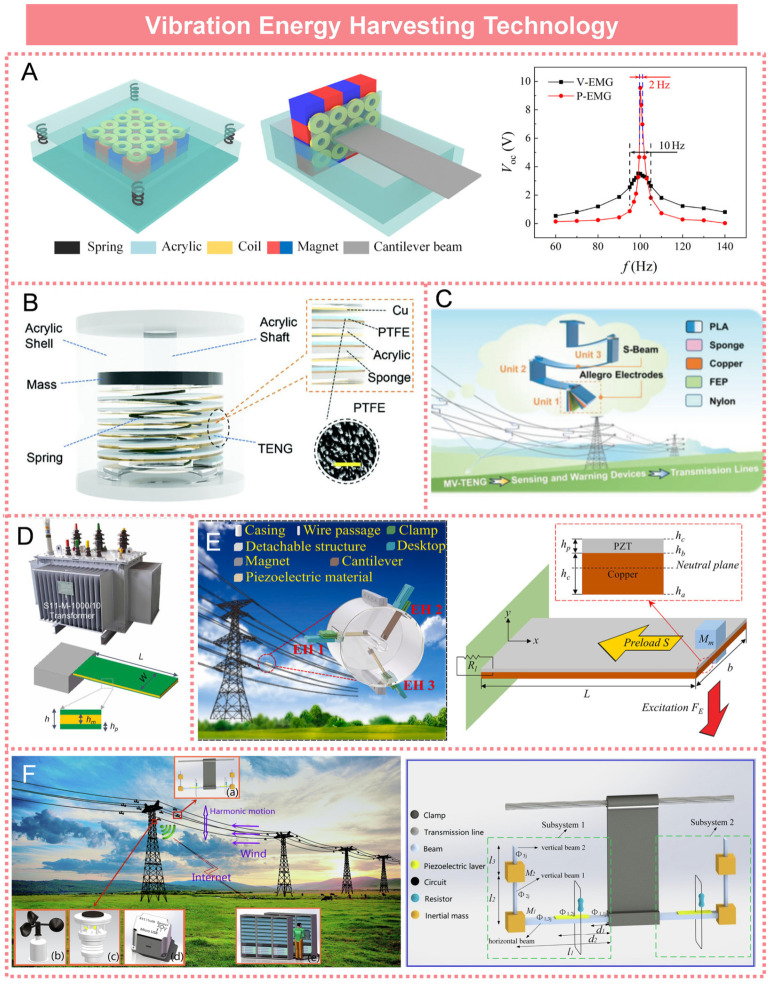
Vibration energy harvesting technologies: (**A**) Electromagnetic vibration energy harvesting device based on alternating magnet arrays. Reprinted with permission from [105]. (**B**) Self-powered sensing network based on triboelectric nanogenerator. Reprinted with permission from [106]. (**C**) Multimodal vibration triboelectric nanogenerator. Reprinted with permission from [107]. (**D**) Self-powered transformer temperature monitoring system. Reprinted with permission from [108]. (**E**) High-performance piezoelectric vibration energy harvester. Reprinted with permission from [109]. (**F**) Novel broadband piezoelectric energy harvester based on internal resonance. Reprinted with permission from [110].

Lv Pinlei et al. developed an electromagnetic vibration energy harvesting device utilizing an alternating magnet array to capture 100 Hz vibration energy in power grid environments, as illustrated in Figure 9A. Under a vibration frequency of 100 Hz, the device achieved a maximum output power of 15 mW [105]. Due to the significant electromagnetic interference present in power environments, the adoption of electromagnetic conversion mechanisms for vibration energy harvesting is not considered an optimal solution. In recent years, researchers have increasingly favored the development and study of alternative devices that do not produce or interfere with electromagnetic fields. Han Wu et al. developed a self-powered sensor network utilizing triboelectric nanogenerators, as illustrated in Figure 9B. This network, deployed in a distributed configuration along a simulated transmission line, enabled early warning of abnormal vibrations across the entire line as well as real-time monitoring of vibration distribution [106]. Xiaosong Zhang et al. proposed a novel multimodal triboelectric nanogenerator designed for broadband energy harvesting in smart transmission lines, as illustrated in Figure 9C. This device was capable of harvesting vibration energy within the frequency ranges of 1–3.5 Hz in the horizontal direction and 4 Hz, 9–60 Hz in the vertical direction, thereby effectively covering the typical frequency ranges of micro-wind-induced vibrations and transmission line oscillations [107]. Fayang Wang et al. developed an ultra-low acceleration piezoelectric vibration energy harvester for self-powered temperature monitoring of transformers, as illustrated in Figure 9D. This device was capable of generating an output voltage of 0.7 V at an acceleration level of 0.02 g, demonstrating a significantly lower startup acceleration compared to other bimorph piezoelectric energy harvesters [108]. Shanghao Gu et al. developed a high-performance piezoelectric energy harvesting system with anti-interference capabilities, specifically designed for smart grid monitoring, as illustrated in Figure 9E. This device efficiently harvested energy through magnetic coupling between the magnet located at the tip of the piezoelectric cantilever and the alternating magnetic field generated around the power line [109]. Xiaochun Nie et al. proposed a novel broadband piezoelectric energy harvester based on internal resonance for application in smart transmission line systems, as illustrated in Figure 9F. This device was capable of suppressing vortex-induced vibrations in transmission lines across a wind speed range of 1 to 4 m per second and can provide power for online monitoring equipment [110].

Most electrical equipment exhibits stable vibrations during operation, presenting a significant opportunity for the development of vibration energy harvesting technologies. However, current vibration energy harvesting technologies remain in the exploratory phase, and the reliability of associated devices does not yet meet the practical requirements of power systems.

## 7. Multi-Energy Composite Technology

A single energy harvesting technology often struggles to adapt to complex and variable power environments. Consequently, the simultaneous utilization of multiple energy sources has attracted increasing attention from researchers. Composite energy harvesting systems are capable of capturing two or more forms of energy concurrently, offering advantages such as higher power density and improved power supply reliability.

**Figure 10:**
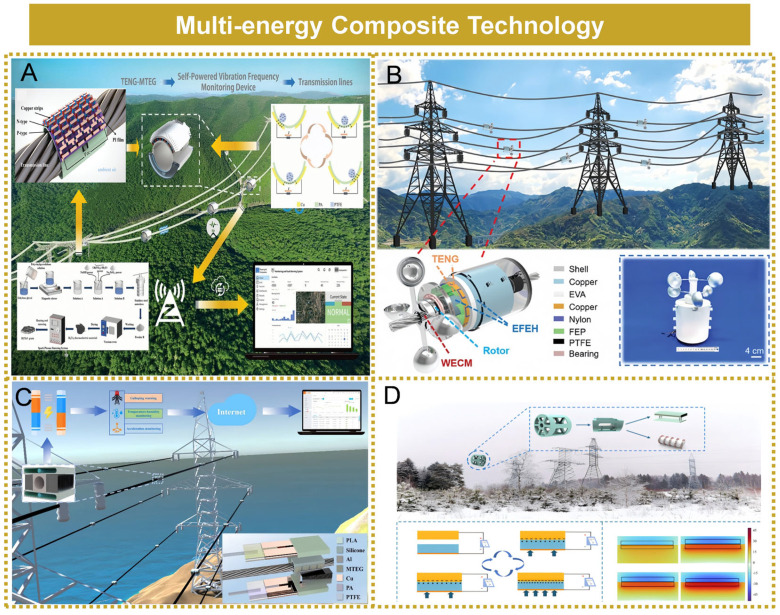
Multiple energy composite technologies. (**A**) Thermal energy and vibration energy composite. Reprinted with permission from [111]. (**B**) Wind energy and electric field energy composite. Reprinted with permission from [112]. (**C**) Thermal energy and vibration energy composite. Reprinted with permission from [113]. (**D**) Thermal energy and vibration energy composite. Reprinted with permission from [114].

Zhijie Hao et al. developed a self-powered grid vibration frequency monitoring device utilizing triboelectric nanogenerators and micro thermoelectric generators [111]. A vibration frequency sensing model based on triboelectric generation and an energy harvesting model based on thermoelectric generation were constructed, as illustrated in Figure 10A. The experimental results indicated that within the frequency range of 0.1 to 5.1 Hz, the triboelectric nanogenerator can accurately detect the vibration frequency of transmission lines, with a maximum measurement error of 1.274%. Additionally, the thermoelectric generator achieved a maximum open-circuit voltage of 3.282 V. Xiaolong Huang et al. developed a triboelectric–electrostatic hybrid energy harvester by integrating a triboelectric nanogenerator with an electric field energy harvester [112], enabling the simultaneous capture of wind energy and ambient electric field energy near transmission lines, as illustrated in Figure 10B. The wind energy harvesting component achieved an output power of 14.3 mW, while the electric field energy harvesting component delivered an output power of 28.9 mW. Xing Feng et al. proposed a hybrid energy harvesting approach that integrated a micro thermoelectric generator with a triboelectric nanogenerator [113] and developed a corresponding theoretical model for the hybrid system, as illustrated in Figure 10C. The prototype was capable of not only capturing energy from the vibration of transmission lines but also converting the thermal energy generated by the lines into electrical energy. Experimental results demonstrated that the hybrid energy harvesting device achieved a 5.36% improvement in both energy harvesting efficiency and battery charging performance. Yingli Lu et al. proposed a self-powered monitoring system for assessing both the thickness and growth kinetics of ice accumulation on transmission lines, utilizing a triboelectric nanogenerator (TENG) and a micro thermoelectric generator (MTEG) [114], as illustrated in Figure 10D. Experimental results indicated that the triboelectric generation module was capable of accurately measuring ice layer thickness within the range of 10 mm to 20 mm, with a maximum measurement error of only 2.14%. Furthermore, it effectively monitored ice growth rates ranging from 0.02 mms^−1^ to 1 mms^−1^, exhibiting a maximum error of 3.65%. The thermoelectric generation unit achieved a peak output voltage of 1.15 V and a maximum current of 180 mA.

Although integrating multiple energy harvesting methods can enable complementarity among different energy sources and improve system reliability, much of the current research primarily focuses on directly combining multiple energy harvesting devices [41,115]. This approach often leads to bulky and complex system designs. Furthermore, a universally effective strategy for achieving synergistic integration across diverse energy sources has yet to be developed.

## 8. Multiple Physical Mechanisms Composite Technology

**Figure 11:**
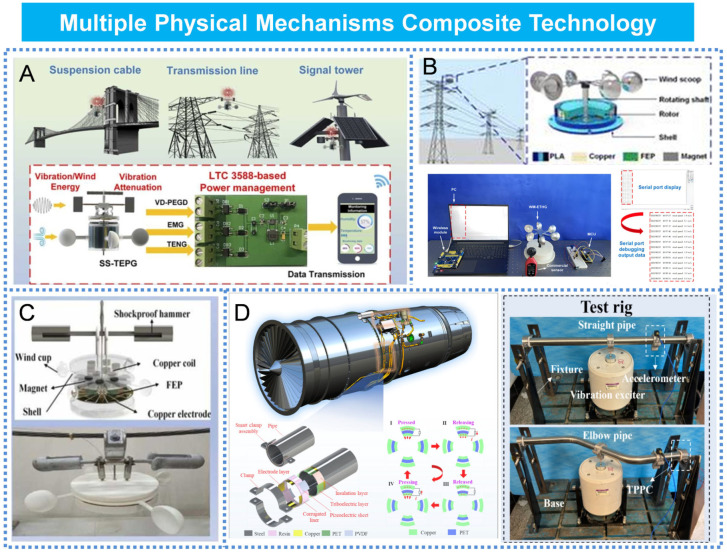
Composite of multiple physical mechanisms: (**A**) Triboelectric–electromagnetic-piezoelectric composite. Reprinted with permission from [121]. (**B**) Triboelectric–electromagnetic composite. Reprinted with permission from [122]. (**C**) Triboelectric–electromagnetic composite. Reprinted with permission from [123]. (**D**) Triboelectric–piezoelectric composite. Reprinted with permission from [124].

The same form of energy can be converted through various physical mechanisms. Consequently, the utilization of multiple physical mechanisms for energy acquisition has garnered increasing attention from researchers [116,117,118,119,120]. Sihang Gao et al. developed a triboelectric–electromagnetic–piezoelectric hybrid energy harvester with a suspended structure, enabling the simultaneous and efficient capture of wind and vibration energy, as illustrated in Figure 11A. Following structural optimization, the vibration energy harvester achieved an instantaneous output power of 8.2 mW, while the TENG and EMG modules of the wind energy harvester delivered instantaneous powers of 1.1 mW and 23.5 mW, respectively [121]. Jing Zhao et al. developed an electromagnetic–triboelectric hybrid nanogenerator for monitoring wind-induced vibrations in transmission lines [122], as illustrated in Figure 11B. Through a well-designed structural configuration, the upper triboelectric nanogenerator and the lower electromagnetic generator were capable of operating concurrently without mutual interference. Sihang Gao et al. developed a triboelectric–electromagnetic hybrid wind turbine designed for the environmental monitoring of high-voltage transmission lines [123], as illustrated in Figure 11C. The triboelectric unit and electromagnetic unit were capable of generating instantaneous peak powers of 7.9 mW and 85.1 mW, respectively. Qingyu Zhu et al. proposed a compact hybrid generator that combines piezoelectric and triboelectric mechanisms, featuring an ultra-compact architecture tailored for embedded applications. This system, as illustrated in Figure 11D, supports distributed deployment and enables intelligent monitoring in complex environments, offering a practical solution for self-powered sensing in power systems [124].

Compared to a single physical mechanism, integrating multiple physical mechanisms can significantly enhance energy harvesting efficiency; however, it also encounters challenges related to complex power management.

## 9. Energy Management Circuit

**Figure 12:**
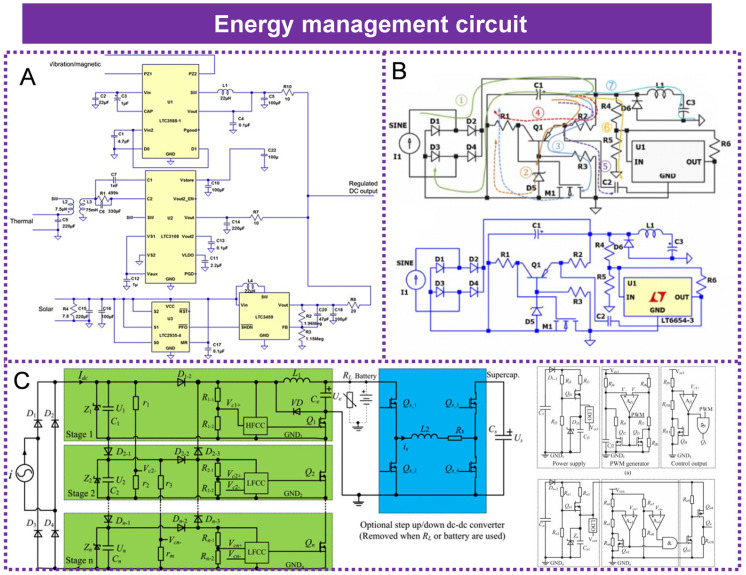
Energy management circuit: (**A**) Scheme of the hybrid energy management circuitry. Reprinted with permission from [125]. (**B**) UVLO circuit design. Reprinted with permission from [126]. (**C**) Cascade ladder-type energy conversion circuit. Note: HFCC: High-frequency control circuit; LFCC: Low-frequency control circuit. Reprinted with permission from [63].

The power management circuit plays an indispensable role in the energy harvesting system. Its core value is to solve the inherent characteristic mismatch between the energy source and the load and to improve the energy conversion and storage efficiency. Feng Yang et al. proposed a hybrid energy harvesting system that integrates magnetic energy, thermoelectric energy, and vibration energy, aiming to provide stable and reliable energy for low-power sensors in the power grid. Through numerical simulation and laboratory experiments, the system evaluated the energy conversion efficiency of the three energy harvesting methods and designed a low-power hybrid energy management system (as shown in Figure 12A) to optimize the overall energy output and ensure that the three energy sources can complement each other and supply power in different environmental conditions. The experimental results show that the output power of the magnetic energy harvesting system can reach 0.7 mW to 366 mW within the current range of 100 A to 800 A; the output power of the thermoelectric energy harvesting system is 12.9 mW to 1.98 W; and the output power of the vibration energy harvesting system is 0.63 mW. The hybrid energy management system achieves a stable 3.3 V DC output by effectively integrating the three energy sources, providing continuous power support for sensors [125]. Ke Zhou et al. proposed a self-powered wireless sensor system based on electric field energy harvesting and designed a low-cost self-driven under-voltage lockout (UVLO) circuit, as shown in Figure 12B, to enhance the efficiency of energy harvesting and storage. They developed a high-voltage UVLO circuit based on voltage-regulating diodes and voltage-monitoring chips. This circuit can monitor the voltage of the energy storage capacitor by adjusting the upper and lower threshold voltages. When the voltage reaches the set upper limit, the circuit locks and starts to supply power to the back-end circuit; when the voltage drops to the set lower limit, the circuit relocks, stops supplying power, and continues charging. This mechanism can effectively manage and optimize the use of energy, ensuring the stable operation of wireless sensor nodes in high-voltage environments. Additionally, by combining multiple-level circuits, the efficiency of energy harvesting and utilization can be further improved. This design not only increases the flexibility of the circuit but also allows for the adjustment of voltage thresholds according to actual needs, making it highly practical [126]. Xiangjun Zeng et al. proposed a power supply solution based on electric field energy harvesting (EFEH), as shown in Figure 12C, aiming to address the power supply issue of high-voltage transmission line monitoring equipment. In this solution, a cascaded trapezoidal topology energy conversion circuit was designed. By increasing the number of cascaded stages, the output power can be significantly enhanced. Experimental results demonstrated that under an input current of 200 μA, the energy harvesting capacity of the four-stage cascaded circuit reached 620 μW/μA, outperforming existing research achievements. To verify the performance of the energy harvester and power circuit, the research team established a high-voltage experimental platform. Experimental data indicated that the actual energy harvesting capacity of the collector was close to the simulation prediction, and the average conversion efficiency of the new circuit was 61%, fully validating its high efficiency and practical application value [63].

## 10. Summary and Outlook

In recent years, energy harvesting devices have experienced rapid development within power systems, highlighting their growing significance in this domain. This paper provides a comprehensive overview of several contemporary energy harvesting techniques applicable to power systems. The advantages and limitations of each method are summarized in Table 5.

**Figure 13:**
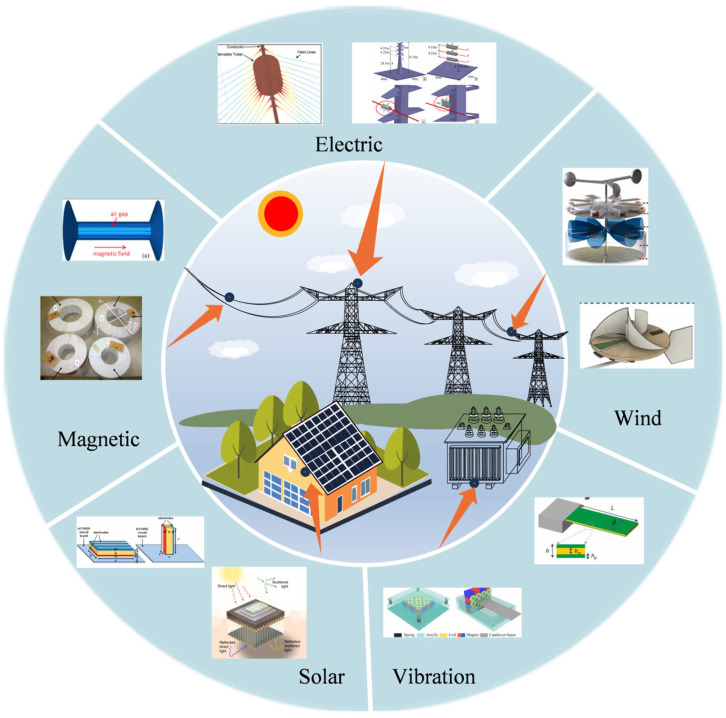
Typical application scenarios. Reprinted with permission from [38,42,62,63,83,84,88,89,105,108].

Magnetic field energy harvesting technology primarily utilizes mutual inductance for energy collection, offering advantages such as a simple structure, stable output voltage, and high power delivery. However, this method imposes strict requirements on the stability of the current in the power line. In the event of a sudden surge in current, the secondary-side conversion circuit and the magnetic core may be susceptible to damage. Electric field energy harvesting technology features a relatively simple structure; however, its power output is significantly limited. To fulfill practical application requirements, the spatial capacitor must be of considerable size, which complicates installation and fixation. Moreover, it may interfere with the insulation clearance of power facilities. Small-scale wind energy harvesting devices have exhibited notable potential in advancing localized energy supply systems. Nevertheless, their efficiency across varying wind directions and a broad spectrum of wind speeds is still constrained, and their structural durability for prolonged outdoor operation requires further improvement. Solar power generation technology offers considerable economic and environmental advantages and has reached a relatively mature stage in terms of technological development. However, its application is primarily limited to outdoor areas with ample sunlight. Furthermore, solar panels are typically large and heavy, and their photovoltaic conversion efficiency remains relatively low. In addition, their performance is significantly compromised under adverse weather conditions, such as cloudy or rainy days, and they are unable to generate power during night-time. The current vibration energy harvesting technology remains in the exploratory phase, and the reliability of associated devices has not yet reached the practical requirements of power systems. Multi-energy composite energy harvesting technology enables complementary utilization among various energy sources and exhibits relatively high power density. Figure 13 visually illustrates the practical implementation of these technologies across various components of the power system. Magnetic field energy harvesting technology is applicable to electrical power transmission and distribution equipment or transmission lines. It generates induced electrical energy through the alternating magnetic field around it, and it is a non-contact energy acquisition method. Electric field energy harvesting technology is mainly used in high-voltage overhead transmission lines. It acquires energy by coupling with the strong electric field around the conductors and has high environmental adaptability. Wind energy harvesting technology is usually deployed on high-voltage transmission towers or sections of lines with high wind speeds. It is suitable for applications such as wind speed measurement and meteorological monitoring, and it can provide continuous and stable energy support for outdoor sensor nodes. Solar energy harvesting technology is widely used in Internet of Things devices in transmission lines, especially in areas with strong outdoor light. It can use reflected light from the ground and buildings as supplementary light sources to improve energy harvesting efficiency. Vibration energy harvesting technology is typically installed on transformers or transmission lines. It can convert mechanical vibrations (such as conductor galloping and equipment noise) into electrical energy and is suitable for high-frequency vibration environments. It can provide self-powering capabilities for vibration monitoring sensors.

To quantitatively evaluate these deployment strategies, Table 6 systematically compares the core performance metrics of each technology, including power density, output power, operational parameters, and application scenarios.

However, the current approach of direct integration results in excessively large device dimensions, complicating the power management of multiple energy sources. The method of combining multiple physical mechanisms can effectively enhance the energy harvesting efficiency compared to a single physical mechanism, but it also faces the bottleneck problem of difficult power management. The future development prospects of energy harvesting technology in power systems are as follows:

(1) Structural design is progressively evolving toward miniaturization and customization to better meet the on-site power supply requirements of electrical equipment.
(2) From the perspective of energy diversification, the trend is shifting toward diversification, integration, and coupling, which can effectively enhance power generation capacity. This evolution is expected to drive significant growth in research related to multi-energy management.
(3) The packaging technology of energy harvesters plays a critical role in ensuring their efficient and reliable performance under diverse environmental conditions, encompassing various design aspects such as mechanical support and protection against environmental degradation. Wang Fei et al. integrated silicon tip arrays into MEMS-based energy harvesters, thereby enabling charge restoration without compromising the integrity of the package [129]. This innovative approach effectively addresses challenges posed by harsh industrial environments, such as elevated humidity and temperature levels, and significantly improves the long-term operational stability of the devices. In the future, researchers should focus on innovations in materials, structures, and system integration to ensure that energy harvesting devices can operate stably over extended periods in harsh environments, thereby providing robust support for the deployment of self-powered sensors in specialized application scenarios.
(4) The interface between energy harvesters and IoT platforms encompasses both hardware integration and data communication protocols, playing a critical role in ensuring that the collected environmental energy—such as solar, wind, or vibrational energy—is efficiently converted into electrical energy and seamlessly integrated into the IoT system. The hardware interface layer encompasses the physical connectivity of energy harvesting devices. In the future, energy harvesters should be directly integrated with the platform via standardized interfaces, such as RS-485, Modbus, or wireless communication protocols. Energy storage units are tasked with temporarily storing harvested energy and regulating its output through power management integrated circuits (ICs), thereby enabling plug-and-play functionality for connected devices. The interface of data communication protocols depends on standardized protocols (e.g., MQTT or LoRaWAN) for the transmission of real-time data. Future development trends emphasize the optimization of low-power communication protocols and the synergistic integration of multi-source energy solutions, aiming to enhance the robustness and interoperability of the interface.
(5) Current standardization efforts primarily aim at improving energy harvesting efficiency and enhancing the adaptability of communication protocols to support distributed energy access. Future standardization work will concentrate on establishing engineering application standards, such as plug-and-play specifications, to ensure seamless integration of energy collection devices, sensors, and other equipment into the system platform, thereby supporting the intelligent development of the new power system.

## Figures and Tables

**Table 1:** Classification of different energy form capture mechanisms.

Energy Form	Capture Mechanism
Magnetic Field Energy	Electromagnetic induction Magnetostriction material Machine construction
Electric Field Energy	High-potential region electrode Low-potential region electrode
Wind Energy	Electromagnetic induction Piezoelectric Triboelectric nanogenerator Multi-mechanism coupling
Solar Energy	Photovoltaic Technology
Vibration Energy	Piezoelectric Electromagnetic Triboelectric
Multi-energy Composite	Triboelectric–thermoelectric Triboelectric–electrostatic

**Table 2:** A summary of recent research on direct magnetic field energy harvesting technology.

Type	Frequency Range	Magnetic Field Strength/Conductor Current	Output Power	Power Density	Document
Standalone magnetic field energy harvesting	50 Hz	18 μT	0.3 mW	0.15 μW/cm^3^	[39]
	50 Hz	900 A	116 mW	\	[40]
	50 Hz	11 μT	146.7 mW	103 μW/cm^3^	[38]
	10 Hz–1 K Hz	30 A	0.36 mW	106 μW/cm^3^ (36 μW/g)	[36]
	50 Hz	6.5 μT	4.5 mW	7.28 μW/cm^3^	[37]
	50 Hz	100 A–800 A	3.67 mW	48.9 μW/cm^3^	[41]
Air-gap-free annular magnetic core	60 Hz	21.2 μT	6.32 mW	1.47μW/cm^3^	[42]
	50 Hz	10 A	283 mW	2.34 mW/cm^3^	[32]
	50 Hz	8.3 A	16.8 mW	\	[31]
	50 Hz	60 A 600 A	1.5 W 19.89 W	1.04 W/cm^3^	[43]
Air-gap annular magnetic core	50 Hz	10 A	63.72 mW	22.01 mW/cm^3^	[44]
	50–60 Hz	909 A	\	0.68 mW/cm^3^	[33]
	60 Hz	615 A	55 W	100.2 mW/cm^3^	[34]
	50 Hz	78.6 mT	32.78 mW	\	[35]

**Table 3:** A summary of the research on indirect magnetic field energy harvesting technology.

Structure	Piezoelectric/Magnetostriction Material	Maximum Output Voltage	Maximum Output Power/ Power Density	Frequency of Collection	Document
Cantilever beam stacking structure	PZT-5H Ni	40.3 V (1.8 Hz)	0.87 mW	50 Hz	[46]
Tuning fork structure	PZT-5H ST	100 V (V_PP_)	72 mW	60 Hz	[47]
Cantilever beam laminated structure	PMN-PZT Metglas	36.5 V (100 Hz 0.02 g)	1.25 mW	60 Hz	[53]
Clamp cantilever beam structure	PZT Copper	1.775 V	970 μW	50 Hz	[54]
Imitating dragonfly structure	PZT TC4	45.5 V (V_PP_)	4.45 mW	50.5 Hz	[55]
-	PZT-5H Fe-Ga	4.58 V	897 μW	50 Hz	[56]
-	CoFe_2_O_4_ NiFe_2_O_4_	12.2 V	243 μW/m^2^	50 Hz	[48]
-	CTFO-BCZCT-CTFO	800 mV/(cm Oe)	-	954 Hz	[57]
-	PMN-PZT Ni	9.52 V(V_pp_)	46.3 mW cm^−3^ Oe^−2^	60 Hz	[58]
-	Fe_52_–Co_48_ alloys	150 mV	34.0 µW·cm^−3^	293 Hz	[49]

**Table 4:** A summary of the technology related to electric field energy harvesting.

Type	Voltage Level	Average Power	μW/μA	Document
High-potential energy harvesting technology	150 kV	370 mW	413 μW/μA	[62]
	110 kV	124 mW	620 μW/μA	[63]
	10 kV	110 mW	3.26 μW/μA	[64]
	35 kV	17 mW	\	[65]
	12.7 kV	23.6 mW	138.8 μW/μA	[60]
	50 kV	340 mW	18.38 μW/μA	[59]
	10 kV	30.7 mW	5.54μW/μA	[66]
Low-potential energy harvesting technology	765 kV	0.17 mW	\	[67]
	110 kV	0.26 mW	25.4 μW/μA	[68]
	230 V	367.5 μW	105 μW/μA	[69]
	110 V/230 V	0.11–1.09 μW	\	[70]
	120 V	2–2.5 µW	\	[71]
	230 V	21.42 μW	25 μW/μA	[72]
	220 V	0.6 μW	\	[73]
	110 kV	48.3 mW	956 μW/μA	[61]

**Table 5:** The advantages and limitations of energy harvesting techniques in power systems.

Type	Strengths	Weaknesses
Magnetic field energy harvesting technology	Simple structure Stable output High output power	High requirements for current Vulnerable to shock Prone to damage
Electric Field Energy Harvesting Technology	Simple structure Non-invasive	Low power Large size Difficult installation
Wind Energy Harvesting Technology	In situ energy supply Non-invasive Wide	Poor stability Difficulties in omnidirectional collection
Solar Energy Harvesting Technology	High maturity High output power	Heavy in weight Large in volume Greatly affected by the environment
Vibration Energy Harvesting Technology	Simple structure Light weight Easy to array	Prone to fatigue Poor reliability

**Table 6:** Quantitative metrics for different energy collection methods.

Type	Power Density	Applicable Environmental Parameters	Usage Scenarios	Document
Magnetic Field Energy Harvesting Technology	1–200 mW/cm^3^	10–1000 Hz (frequency of magnetic field variation)	Electrical transmission and distribution equipment Power transmission lines Electrical appliances	[38,39,47]
Electric Field Energy Harvesting Technology	100–1000 μW/μA	10–1000 kV (transmission line voltage)	High-Voltage Overhead Power Lines High-voltage transmission line Power transmission lines	[61,62,63]
Wind Energy Harvesting Technology	10–200 mW/cm^3^	10–20 m/s (wind speed)	High-voltage transmission lines Transmission tower Wind speed measurement	[83,84,85]
Solar Energy Harvesting Technology	<100 mW/cm^2^	AM1.5 G	Power transmission lines Internet of Things applications Outdoor concrete ground	[86,88,89,127]
Vibration Energy Harvesting Technology	10–200 μW/cm^3^	10–100 Hz (vibration frequency)	Transformer Power transmission lines	[105,107,108,128]

## References

[1] Evans D. (2011). The internet of things. how the next evolution of the internet is changing everything, whitepaper. Cisco Internet Bus. Solut. Group (IBSG).

[2] Elbouchikhi E., Zia M.F., Benbouzid M., El Hani S. (2021). Overview of Signal Processing and Machine Learning for Smart Grid Condition Monitoring. Electronics.

[3] Yilmaz S., Dener M. (2024). Security with Wireless Sensor Networks in Smart Grids: A Review. Symmetry.

[4] Ma J., Sun H., Chu Z., Zhao J., Yang Z., Liu S., Wen J., Qin Y. (2025). Self-powered and self-calibrated sensing system for real-time environmental monitoring. Sci. Adv..

[5] Gholikhani M., Roshani H., Dessouky S., Papagiannakis A.T. (2020). A critical review of roadway energy harvesting technologies. Appl. Energy.

[6] Han D.Y., Song C.K., Lee G., Song W.J., Park S. (2024). A Comprehensive Review of Battery-Integrated Energy Harvesting Systems. Adv. Mater. Technol..

[7] Luo H., Yang T., Jing X., Cui Y., Qin W. (2024). Environmental energy harvesting boosts self-powered sensing. Mater. Today Energy.

[8] Zhang D., Zhou L., Wu Y., Yang C., Zhang H. (2024). Triboelectric Nanogenerator for Self-Powered Gas Sensing. Small.

[9] Jiang W., Han X., Chen L., Bi Q. (2020). Bursting vibration-based energy harvesting. Nonlinear Dyn..

[10] Zhu D. (2022). Advance Energy Harvesting Technologies. Energies.

[11] Chong Y.W., Ismail W., Ko K., Lee C.Y. (2019). Energy Harvesting For Wearable Devices: A Review. IEEE Sens. J..

[12] Babayo A.A., Anisi M.H., Ali I. (2017). A Review on energy management schemes in energy harvesting wireless sensor networks. Renew. Sustain. Energy Rev..

[13] Luo A., Tan Q., Xu W., Huang J., Gu S., Guo X., Lee C., Fan K., Wang F. (2025). A Comprehensive Review of Energy Harvesting From Kinetic Energy at Low Frequency. Adv. Mater. Technol..

[14] Zhang H., Shen Q., Zheng P., Wang H., Zou R., Zhang Z., Pan Y., Zhi J.Y., Xiang Z.R. (2024). Harvesting inertial energy and powering wearable devices: A review. Small Methods.

[15] Junior O.A., Maran A., Henao N. (2018). A review of the development and applications of thermoelectric microgenerators for energy harvesting. Renew. Sustain. Energy Rev..

[16] Espe A.E., Haugan T.S., Mathisen G. (2022). Magnetic field energy harvesting in railway. IEEE T Power Electr..

[17] Seo D., Kim B., Ha M., Han S., Yoon S., Cho B. (2025). Energy harvesting with magneto-mechano-electric harvester for AC circular magnetic fields. Sens. Actuat. A-Phys..

[18] Riba J.-R., Arbat R., Ndong Y.O., Moreno-Eguilaz M. (2023). Exploring the Limitations of Electric Field Energy Harvesting. Electronics.

[19] Menéndez O., Villacrés J., Prado A., Vásconez J.P., Auat-Cheein F. (2024). Assessment of Triboelectric Nanogenerators for Electric Field Energy Harvesting. Sensors.

[20] Li X., Hu G., Guo Z., Wang J., Yang Y., Liang J. (2022). Frequency up-conversion for vibration energy harvesting: A review. Symmetry.

[21] Qi L., Pan H., Pan Y., Luo D., Yan J., Zhang Z. (2022). A review of vibration energy harvesting in rail transportation field. Iscience.

[22] Zheng X., He L., Wang S., Liu X., Liu R., Cheng G. (2023). A review of piezoelectric energy harvesters for harvesting wind energy. Sens. Actuat. A-Phys..

[23] Ali A., Ali S., Shaukat H., Khalid E., Behram L., Rani H., Altabey W.A., Kouritem S.A., Noori M. (2024). Advancements in piezoelectric wind energy harvesting: A review. Results Eng..

[24] Satharasinghe A., Hughes-Riley T., Dias T. (2020). A review of solar energy harvesting electronic textiles. Sensors.

[25] Massiot I., Cattoni A., Collin S. (2020). Progress and prospects for ultrathin solar cells. Nat. Energy.

[26] Yang J., Zhang W., Zou L., Wang Y., Sun Y., Feng Y. (2019). Research on distribution and shielding of spatial magnetic field of a DC air core smoothing reactor. Energies.

[27] Liu L., Wen X., Shi R., Li P., Wen Y., Han T. (2023). High-efficiency magnetic field energy harvesting from a three-core cable. Sens. Actuat. A-Phys..

[28] Tao J., Wang Y., Cheng H., Mai R. (2023). Analysis and Design of Magnetic Field Energy Harvesting for Freight Train Sensors. IEEE Trans. Transp. Electrif..

[29] Bhuiyan R.H., Dougal R.A., Ali M. (2010). A miniature energy harvesting device for wireless sensors in electric power system. IEEE Sens. J..

[30] Park B., Kim D., Park J., Kim K., Koo J., Park H., Ahn S. (2018). Optimization design of toroidal core for magnetic energy harvesting near power line by considering saturation effect. AIP Adv..

[31] Gaikwad A., Kulkarni S. (2018). Evaluation of dimensional effect on electromagnetic energy harvesting. Procedia Comput. Sci..

[32] Zhuang Y., Xu C., Song C., Chen A., Lee W., Huang Y., Zhou J. (2019). Improving current transformer-based energy extraction from AC power lines by manipulating magnetic field. IEEE Trans. Ind. Electron..

[33] Paul S., Bashir S., Chang J. (2018). Design of a novel electromagnetic energy harvester with dual core for deicing device of transmission lines. IEEE Trans. Magn..

[34] Najafi S.A.A., Ali A.A., Sozer Y., De Abreu-Garcia A. Energy harvesting from overhead transmission line magnetic fields. Proceedings of the 2018 IEEE Energy Conversion Congress and Exposition (ECCE).

[35] Zhang J., Tian X., Li J., Yan D. (2020). A novel electromagnetic energy harvester based on double-ring core for power line energy harvesting. J. Circuit. Syst. Comp..

[36] Wright S.W., Kiziroglou M.E., Spasic S., Radosevic N., Yeatman E.M. (2019). Inductive energy harvesting from current-carrying structures. IEEE Sens. Lett..

[37] Wang H., Shi G., Han C., Monti G. (2021). A free-standing electromagnetic energy harvester for condition monitoring in smart grid. Wirel. Power Transf..

[38] Yuan S., Huang Y., Zhou J., Xu Q., Song C., Thompson P. (2015). Magnetic field energy harvesting under overhead power lines. IEEE T Power Electr..

[39] Roscoe N.M., Judd M.D. (2013). Harvesting energy from magnetic fields to power condition monitoring sensors. IEEE Sens. J..

[40] Moghe R., Lambert F.C., Divan D. (2011). Smart “stick-on” sensors for the smart grid. IEEE Trans. Smart Grid.

[41] Li Q., Zhang L., Zhang C., Tian Y., Fan Y., Li B., An Z., Li D., Wang Z.L. (2024). Compact, robust, and regulated-output hybrid generators for magnetic energy harvesting and self-powered sensing applications in power transmission lines. Energ. Environ. Sci..

[42] Tashiro K., Wakiwaka H., Inoue S.-I., Uchiyama Y. (2011). Energy harvesting of magnetic power-line noise. IEEE Trans. Magn..

[43] Muñoz-Gómez A.-M., Menéndez-Marín M., Ballestín-Fuertes J., Sanz-Osorio J.-F. (2025). Single-Stage Power Converter for Magnetic Field Energy Harvesting to Achieve Self-Powered Smart Grid IoT Devices. Electronics.

[44] Wu Z., Wen Y., Li P. (2013). A power supply of self-powered online monitoring systems for power cords. IEEE Trans. Energy Convers..

[45] Yu Y., Cheng Z., Chang J., Mai Z., Wang B., Zhu R., Sun M., Dong S., Ci P. (2024). Enhanced In-Plane Omnidirectional Energy Harvesting from Extremely Weak Magnetic Fields via Fourfold Symmetric Magneto-Mechano-Electric Coupling. Adv. Energy Mater..

[46] He X., Xu Y., Wu J., Huang H., Liang X., Du Y., Qiao J., Li Y., Huang H., Ju D. (2025). Enhanced Power Density by Resonant Frequency Optimization in Magneto-Mechano-Electric Generator for Multifunctional Wireless Sensor System. Small.

[47] Yu Z., Qiu H., Chu Z., Sun Z., Asl M.J.P., Li F., Dong S. (2022). Significant output power enhancement in symmetric dual-mode magneto-mechano-electric coupled resonators. Adv. Energy Mater..

[48] Pabba D.P., Kaarthik J., Ram N., Venkateswarlu A. (2024). Harnessing the Induced Magnetostrictive Effect in Fully Flexible Fiber-Based Magnetoelectric Composites for Improved Stray Magnetic Energy Harvesting. ACS Appl. Electron. Mater..

[49] Kurita H., Lohmuller P., Laheurte P., Nakajima K., Narita F. (2022). Additive manufacturing and energy-harvesting performance of honeycomb-structured magnetostrictive Fe52–Co48 alloys. Addit. Manuf..

[50] Jin X., Yuan Z., Shi Y., Sun Y., Li R., Chen J., Wang L., Wu Z., Wang Z.L. (2022). Triboelectric nanogenerator based on a rotational magnetic ball for harvesting transmission line magnetic energy. Adv. Funct. Mater..

[51] Yuan Z., Wei X., Jin X., Sun Y., Wu Z., Wang Z.L. (2021). Magnetic energy harvesting of transmission lines by the swinging triboelectric nanogenerator. Mater. Today Energy.

[52] Yu Z., Yang J., Xu L., Chang J., Li Z., Yuan X., Dong S. (2024). Giant tridimensional power responses in a T-shaped magneto–mechano–electric energy harvester. Energ. Environ. Sci.

[53] Sriramdas R., Kang M.G., Meng M., Kiani M., Ryu J., Sanghadasa M., Priya S. (2020). Large power amplification in magneto-mechano-electric harvesters through distributed forcing. Adv. Energy Mater..

[54] Chu Z., Sun Z., Wang B., Song K., Wang J., Gao J., Dong S. (2022). Significantly enhanced power generation from extremely low-intensity magnetic field via a clamped-clamped magneto-mechano-electric generator. Adv. Energy Mater..

[55] Chang J., Gao X., Peng W., Yu Z., Chu Z., Gao J., Liu M., Ci P., Dong S. (2023). A dragonfly-wing-like energy harvester with enhanced magneto-mechano-electric coupling. Device.

[56] Liu J., He Z., Mi C., Sha Y., Zhu X., Hao H., Chen L., Zuo L. (2024). Enhancement of magnetoelectric coupling in laminate composites of textured Fe–Ga thin sheet and PZT. AIP Adv..

[57] Praveen J.P., Monaji V.R., Chandrakala E., Indla S., Subramanian V., Das D. (2018). Enhanced magnetoelectric coupling in Ti and Ce substituted lead free CFO-BCZT laminate composites. J. Alloys Compd..

[58] Ryu J., Kang J.-E., Zhou Y., Choi S.-Y., Yoon W.-H., Park D.-S., Choi J.-J., Hahn B.-D., Ahn C.-W., Kim J.-W. (2015). Ubiquitous magneto-mechano-electric generator. Energ. Environ. Sci.

[59] Li Z., Mei H., Wang L. (2019). A power supply technology for a low-power online monitoring sensor based on electric field induction. Sensors.

[60] Rodriguez J.C., Holmes D.G., Mcgrath B., Wilkinson R.H. (2017). A self-triggered pulsed-mode flyback converter for electric-field energy harvesting. IEEE J. Emerg. Sel. Top. Power Electron..

[61] Hu D., Wang Q., Zheng D., Huang X., Wu Z., Wang F., Xu S., Chen S. (2024). Highly efficient harvesting of electric-field energy from Maxwell’s displacement current by managing charge transfer. Nano Energy.

[62] Zangl H., Bretterklieber T., Brasseur G. (2009). A feasibility study on autonomous online condition monitoring of high-voltage overhead power lines. IEEE Trans. Instrum. Meas..

[63] Zeng X., Yang Z., Wu P., Cao L., Luo Y. (2020). Power source based on electric field energy harvesting for monitoring devices of high-voltage transmission line. IEEE Trans. Ind. Electron..

[64] Zhang J., Li P., Wen Y., Zhang F., Yang C. (2015). A management circuit with upconversion oscillation technology for electric-field energy harvesting. IEEE T Power Electr..

[65] Moghe R., Iyer A.R., Lambert F.C., Divan D. (2014). A low-cost electric field energy harvester for an MV/HV asset-monitoring smart sensor. IEEE Trans. Ind. Appl..

[66] Zhou L., Liu H., Zuo K., Shang K. (2024). Energy harvesting technology for AC overhead insulated transmission line based on electric field induction. High Volt..

[67] Kang S., Kim J., Yang S., Yun T., Kim H. (2017). Electric field energy harvesting under actual three-phase 765 kv power transmission lines for wireless sensor node. Electron. Lett..

[68] Hu D., Huang X., Zheng D., Wu Z., Ding C., Wang F., Xu S., Chen S. (2024). Hybrid nanogenerator for harvesting electric-field and vibration energy simultaneously via Maxwell’s displacement current. Nano Energy.

[69] Zhou J., Zhang J., Xu C., Fang L., Wang Y., Zhuang Y., Jia T., Huang Y., Han C. (2022). On the improvement of electric field energy harvesting from domestic power lines. AEU-Int. J. Electron. Commun..

[70] Gulati M., Parizi F.S., Whitmire E., Gupta S., Ram S.S., Singh A., Patel S.N. (2018). CapHarvester: A stick-on capacitive energy harvester using stray electric field from AC power lines. Proc. ACM Interact. Mob. Wearable Ubiquitous Technol..

[71] Khan M.R., Islam M.A., Rana M.M., Haque T., Joy S.I.I. (2021). A Circuit Model for Energy Harvesting from Fringing Electric Fields for Mobile Wearable Device Applications. Energies.

[72] Menéndez O., Romero L., Cheein F.A. (2020). Serial switch only rectifier as a power conditioning circuit for electric field energy harvesting. Energies.

[73] Menéndez O., Kouro S., Pérez M., Cheein F.A. (2019). Mechatronized maximum power point tracking for electric field energy harvesting sensor. AEU-Int. J. Electron. Commun..

[74] Ren Z., Wu L., Pang Y., Zhang W., Yang R. (2022). Strategies for effectively harvesting wind energy based on triboelectric nanogenerators. Nano Energy.

[75] Li Y., Huang M., Tang T., Mei M., Zhao H., Zha F., Sun L., Liu H. (2025). A High-Power Non-Contact Magnetic Conversion-Enhanced Wind Energy Harvester for Self-Powered IoT Nodes and Real-Time Wind Speed Sensing. Nano Energy.

[76] Wang Y., Yang E., Chen T., Wang J., Hu Z., Mi J., Pan X., Xu M. (2020). A novel humidity resisting and wind direction adapting flag-type triboelectric nanogenerator for wind energy harvesting and speed sensing. Nano Energy.

[77] Dai S., Li X., Jiang C., Zhang Q., Peng B., Ping J., Ying Y. (2022). Omnidirectional wind energy harvester for self-powered agro-environmental information sensing. Nano Energy.

[78] Zhao C., Hu G., Yang Y. (2022). A cantilever-type vibro-impact triboelectric energy harvester for wind energy harvesting. Mech. Syst. Signal Process..

[79] Zhang J., Fang Z., Shu C., Zhang J., Zhang Q., Li C. (2017). A rotational piezoelectric energy harvester for efficient wind energy harvesting. Sens. Actuat. A-Phys..

[80] Zhao L., Zou H., Yan G., Liu F., Tan T., Zhang W., Peng Z., Meng G. (2019). A water-proof magnetically coupled piezoelectric-electromagnetic hybrid wind energy harvester. Appl. Energy.

[81] Gao S., Feng S., Wang J., Wu H., Chen Y., Zhang J., Li Y., Wang R., Luo X., Wei H. (2023). Hybridized triboelectric-electromagnetic aeolian vibration generator as a self-powered system for efficient vibration energy harvesting and vibration online monitoring of transmission lines. ACS Appl. Mater. Interfaces.

[82] Gao S., Zeng X., Zhang G., Zhang J., Chen Y., Feng S., Lan W., Zhou J., Wang Z.L. (2022). Triboelectric–electromagnetic hybridized module for energy harvesting of power transmission lines galloping and self-powered galloping state monitoring. Nano Energy.

[83] Gao S., Zeng X., Chen X., Liao T., Wang R., Chen Y., Wei H., Luo X., Feng S. (2023). Self-powered system for environment and aeolian vibration monitoring in the high-voltage transmission system by multi-directional wind-driven triboelectric nanogenerator. Nano Energy.

[84] Tang X., Hou W., Zheng Q., Fang L., Zhu R., Zheng L. (2022). Self-powered wind sensor based on triboelectric nanogenerator for detecting breeze vibration on electric transmission lines. Nano Energy.

[85] Zou H., Zhao L., Wang Q., Gao Q., Yan G., Wei K., Zhang W. (2022). A self-regulation strategy for triboelectric nanogenerator and self-powered wind-speed sensor. Nano Energy.

[86] Sun W., Liu Y., Ma L., Zhang R. (2021). Research on life extension method of transmission line intelligent sensing system based on environmental energy harvesting. J. Sens..

[87] Guo B., Li Q., Jiang W., Lu K., Cheng H. (2023). A Deep Sensing System for Monitoring the Health Status of Transmission Line Equipment Based on Self-Powered Sensors. IEEE Sens. J..

[88] Prakoso A.B., Wang J., Lu C., Wang H. (2020). PV-Tower solar cell for small footprint photovoltaic energy harvesting for the internet of things application. Semicond. Sci. Technol..

[89] De Bastiani M., Mirabelli A.J., Hou Y., Gota F., Aydin E., Allen T.G., Troughton J., Subbiah A.S., Isikgor F.H., Liu J. (2021). Efficient bifacial monolithic perovskite/silicon tandem solar cells via bandgap engineering. Nat. Energy.

[90] Liu C., Jing X. (2016). Vibration energy harvesting with a nonlinear structure. Nonlinear Dyn..

[91] Du Y., Deng J., Li P., Wen Y. (2020). Energy transfer and redistribution: An approach for unifying vibrational energy harvesting and vibration attenuation. Nano Energy.

[92] Li Y., Tao K., George B., Tan Z. (2020). Harvesting vibration energy: Technologies and challenges. IEEE Ind. Electron. Mag..

[93] Yang T., Zhou S., Fang S., Qin W., Inman D.J. (2021). Nonlinear vibration energy harvesting and vibration suppression technologies: Designs, analysis, and applications. Appl. Phys. Rev..

[94] Xiang Z., Zhang J., Li S., Xie S., Liu F., Zhu R., He D. (2023). Friction-induced vibration energy harvesting via a piezoelectric cantilever vibration energy collector. Tribol. Int..

[95] Liu Q., Qin W., Yang T., Deng W., Zhou Z. (2023). Harvesting weak vibration energy by amplified inertial force and super-harmonic vibration. Energy.

[96] Zhang Y., Zhang G., Wang W. (2024). A piezoelectric cantilever-beam-spring-pendulum oscillator for multi-directional vibration energy harvesting. Commun. Nonlinear Sci.

[97] Deng H., Du Y., Wang Z., Ye J., Zhang J., Ma M., Zhong X. (2019). Poly-stable energy harvesting based on synergetic multistable vibration. Commun. Phys..

[98] Su X., Tong C., Pang H., Tomovic M. (2023). Research on pendulum-type tunable vibration energy harvesting. Energy.

[99] Xu J., Tat T., Zhao X., Xiao X., Zhou Y., Yin J., Chen K., Chen J. (2023). Spherical magnetoelastic generator for multidirectional vibration energy harvesting. ACS Nano.

[100] Wei C., Jing X. (2017). A comprehensive review on vibration energy harvesting: Modelling and realization. Renew. Sustain. Energy Rev..

[101] Wang W., Yin N., Wu Z., Zhang Z. (2025). Omnidirectional energy harvesting with 3D-TENG for vibration diagnosis. Chem. Eng. J..

[102] Xu X., Wu Q., Pang Y., Cao Y., Fang Y., Huang G., Cao C. (2022). Multifunctional metamaterials for energy harvesting and vibration control. Adv. Funct. Mater..

[103] Wang X., Yin G., Sun T., Xu X., Rasool G., Abbas K. (2024). Mechanical vibration energy harvesting and vibration monitoring based on triboelectric nanogenerators. Energy Technol..

[104] Liu Z., Zhao C., Hu G., Yang Y. (2023). A multi-degree-of-freedom triboelectric energy harvester for dual-frequency vibration energy harvesting. Mech. Syst. Signal Process..

[105] Lv P., Fan C., Yang A., Yuan H., Chu J., Rong M., Wang X. (2024). Research on vibration energy harvesting technology of power equipment based on alternating magnet array. High Volt..

[106] Wu H., Wang J., Wu Z., Kang S., Wei X., Wang H., Luo H., Yang L., Liao R., Wang Z.L. (2022). Multi-parameter optimized triboelectric nanogenerator based self-powered sensor network for broadband aeolian vibration online-monitoring of transmission lines. Adv. Energy Mater..

[107] Zhang X., Yu Y., Xia X., Zhang W., Cheng X., Li H., Wang Z.L., Cheng T. (2023). Multi-Mode Vibrational Triboelectric Nanogenerator for Broadband Energy Harvesting and Utilization in Smart Transmission Lines. Adv. Energy Mater..

[108] Wang F., Zhou M., Wu P., Gao L., Chen X., Mu X. (2023). Self-powered transformer intelligent wireless temperature monitoring system based on an ultra-low acceleration piezoelectric vibration energy harvester. Nano Energy.

[109] Gu S., Xu W., Xi K., Luo A., Fan K., Wang F. (2024). High-performance piezoelectric energy harvesting system with anti-interference capability for smart grid monitoring. Renew. Energy.

[110] Nie X., Tan T., Yan Z., Yan Z., Zhang W. (2020). Ultra-wideband piezoelectric energy harvester based on Stockbridge damper and its application in smart grid. Appl. Energy.

[111] Hao Z., Liu C., Shao T., Ma Z., Lu Y., Wang Y., Sui Z. (2024). Self-Powered Vibration Frequency Monitoring Device for the Grid Based on Triboelectric Nanogenerator and Micro Thermoelectric Generator. Adv. Sustain. Syst..

[112] Huang X., Hu D., Wang Q., Wu Z., Wang N., Chen Z., Xu S., Chi M., Chen S. (2025). Hybrid Nanogenerator Harvesting Electric-Field and Wind Energy for Self-Powered Sensors on High-Voltage Transmission Lines. Adv. Funct. Mater..

[113] Feng X., Hao Z., Shao T., Ma Z., Lu Y., Wang Y., Liu C. (2024). A hybrid energy harvesting approach for transmission lines based on triboelectric nanogenerator and micro thermoelectric generator. Nanotechnology.

[114] Lu Y., Liu C., Wang Y., Hao Z., Chen C., Dong B., Zhou X. (2025). A self-powered ice growth sensing system for transmission lines based on a triboelectric nanogenerator and a micro thermoelectric generator. Nanoscale.

[115] Wang Q., Hu D., Huang X., Chen Z., Yuan Z., Zhong L., Sun Q., Wang F., Xu S., Chen S. (2025). Hybrid Triboelectric-Electromagnetic-Electric Field Energy Harvester for Simultaneous Wind and Electric Field Energy Capture in High-Voltage Transmission System. Adv. Energy Mater..

[116] Ryu H., Yoon H.-J., Kim S.-W. (2019). Energy harvesters: Hybrid energy harvesters: Toward sustainable energy harvesting. Adv. Mater..

[117] Egbe K.-J.I., Nazar A.M., Jiao P. (2022). Piezoelectric-triboelectric-electromagnetic hybrid rotational energy harvesters (H-REH). Int. J. Mech. Sci..

[118] He L., Han Y., Sun L., Wang H., Zhang Z., Cheng G. (2023). A rotating piezoelectric-electromagnetic hybrid harvester for water flow energy. Energy Convers. Manag..

[119] Zhu Y., Zhang Z., Zhang P., Tan Y. (2022). A magnetically coupled piezoelectric–electromagnetic low-frequency multidirection hybrid energy harvester. Micromachines.

[120] Tang G., Wang Z., Hu X., Wu S., Xu B., Li Z., Yan X., Xu F., Yuan D., Li P. (2022). A non-resonant piezoelectric–electromagnetic–triboelectric hybrid energy harvester for low-frequency human motions. Nanomaterials.

[121] Gao S., Wei H., Wang J., Luo X., Wang R., Chen Y., Xiang M., Chen X., Xie H., Feng S. (2024). Self-powered system by a suspension structure-based triboelectric-electromagnetic-piezoelectric hybrid generator for unifying wind energy and vibration harvesting with vibration attenuation function. Nano Energy.

[122] Zhao J., Han L., Xu Z., Zhang Y., Li X., Pei Y., Zheng R., Yu X., Jiang C. (2023). Self-powered sensor for monitoring wind vibration on transmission lines based on an electromagnetic-triboelectric hybrid generator. Sustain. Energ. Fuels.

[123] Gao S., Luo X., Wei H., Wang R., Chen X., Zhang J. (2024). Wind-driven suspended triboelectric-electromagnetic hybrid generator with vibration elimination for environmental monitoring in the high-voltage power transmission line. Nano Energy.

[124] Zhu Q., Zhu L., Wang Z., Zhang X., Li Q., Han Q., Yang Z., Qin Z. (2025). Hybrid triboelectric-piezoelectric nanogenerator assisted intelligent condition monitoring for aero-engine pipeline system. Chem. Eng. J..

[125] Yang F., Du L., Chen W., Li J., Wang Y., Wang D. (2017). Hybrid energy harvesting for condition monitoring sensors in power grids. Energy.

[126] Zhou K., Wang X., Wang L., Duan C., Zhang Y., Zhao L., Maeda R. (2024). Self-powered wireless sensing system with cylindrical high voltage side electric field energy harvesting by discharge circuit. Sens. Actuat. A-Phys..

[127] Cetinkaya O., Akan O.B. (2017). Electric-Field Energy Harvesting in Wireless Networks. IEEE Wirel. Commun..

[128] Riba J.-R., Moreno-Eguilaz M., Bogarra S. (2022). Energy Harvesting Methods for Transmission Lines: A Comprehensive Review. Appl. Sci..

[129] Li M., Luo A., Luo W., Liu X., Wang F. (2023). Electrostatic Vibration Energy Harvester With a Self-Rechargeable Electret. IEEE Electron. Device Lett..

